# Extracellular Vesicle-Mediated RNA Release in *Histoplasma capsulatum*

**DOI:** 10.1128/mSphere.00176-19

**Published:** 2019-03-27

**Authors:** Lysangela R. Alves, Roberta Peres da Silva, David A. Sanchez, Daniel Zamith-Miranda, Marcio L. Rodrigues, Samuel Goldenberg, Rosana Puccia, Joshua D. Nosanchuk

**Affiliations:** a Instituto Carlos Chagas, Fiocruz, Curitiba, Cidade Industrial de Curitiba, Brazil; b Departamento de Microbiologia, Imunologia e Parasitologia da Escola Paulista de Medicina, Universidade Federal de São Paulo—UNIFESP, São Paulo, Brazil; c Departments of Medicine (Division of Infectious Diseases) and Microbiology and Immunology, Albert Einstein College of Medicine, Bronx, New York, USA; d Instituto de Microbiologia, Universidade Federal do Rio de Janeiro, Rio de Janeiro, Brazil; Carnegie Mellon University

**Keywords:** *Histoplasma capsulatum*, RNA, extracellular vesicles

## Abstract

Eukaryotic cells, including fungi, release extracellular vesicles (EVs). These lipid bilayered compartments play essential roles in cellular communication and pathogenesis. EV composition is complex and includes proteins, glycans, pigments, and RNA. RNAs with putative roles in pathogenesis have been described in EVs produced by fungi. Here we describe the RNA content in EVs produced by the G186AR and G217B strains of Histoplasma capsulatum, an important human-pathogenic fungal pathogen. A total of 124 mRNAs were identified in both strains. In this set of RNA classes, 93 transcripts were enriched in EVs from the G217B strain, whereas 31 were enriched in EVs produced by the G186AR strain. This result suggests that there are important strain-specific properties in the mRNA composition of fungal EVs. We also identified short fragments (25 to 40 nucleotides in length) that were strain specific, with a greater number identified in EVs produced by the G217B strain. Remarkably, the most highly enriched processes were stress responses and translation. Half of these fragments aligned to the reverse strand of the transcript, suggesting the occurrence of microRNA (miRNA)-like molecules in fungal EVs. We also compared the transcriptome profiles of H. capsulatum with the RNA composition of EVs, and no correlation was observed. Taking the results together, our study provided information about the RNA molecules present in H. capsulatum EVs and about the differences in composition between the strains. In addition, we found no correlation between the most highly expressed transcripts in the cell and their presence in the EVs, reinforcing the idea that the RNAs were directed to the EVs by a regulated mechanism.

**IMPORTANCE** Extracellular vesicles (EVs) play important roles in cellular communication and pathogenesis. The RNA molecules in EVs have been implicated in a variety of processes. EV-associated RNA classes have recently been described in pathogenic fungi; however, only a few reports of studies describing the RNAs in fungal EVs are available. Improved knowledge of EV-associated RNA will contribute to the understanding of their role during infection. In this study, we described the RNA content in EVs produced by two isolates of Histoplasma capsulatum. Our results add this important pathogen to the current short list of fungal species with the ability to use EVs for the extracellular release of RNA.

## Abstract (precis)

Extracellular vesicles (EVs) play important roles in cellular communication and pathogenesis. The RNA molecules in EVs have been implicated in a variety of processes. EV-associated RNA classes have recently been described in pathogenic fungi; however, only a few reports of studies describing the RNAs in fungal EVs are available. Improved knowledge of EV-associated RNA will contribute to the understanding of their role during infection. In this study, we described the RNA content in EVs produced by two isolates of Histoplasma capsulatum. Our results add this important pathogen to the current short list of fungal species with the ability to use EVs for the extracellular release of RNA.

## INTRODUCTION

Histoplasma capsulatum is a major human fungal pathogen on the global stage that causes disease in both immunocompetent and immunocompromised individuals, albeit the risk for severe disease increases with compromised immunity (e.g., in patients with HIV infection or cancer as well as in individuals receiving steroids or tumor necrosis factor alpha [TNF-α] blockers). In the United States, it is the most common cause of fungal pneumonia (1). H. capsulatum is of particular concern in certain developing regions (2), especially in Latin American countries, including Brazil (3, 4), Guatemala (5), and French Guiana, where it is considered the “first cause of AIDS-related death” (6). Despite its clear importance, enormous gaps exist in our understanding of the pathogenesis of histoplasmosis, the disease caused by H. capsulatum. An interesting facet of the biology of H. capsulatum is its ability to release extracellular vesicles (EVs) (7, 8).

EVs are bilayered lipid structures released by remarkably diverse cells across all kingdoms (9). We have demonstrated that EVs are present in both ascomycetes and basidiomycetes (7, 10–14). This observation implies that mechanisms for EV production and release are truly ancient, as they appear to predate the divergence of these branches 0.5–1.0 billion years ago. Fungal EVs can carry biologically active proteins, carbohydrates, lipids, pigments and nucleic acids (15, 16), many of which are constituents of the fungal cell wall and diverse others are associated with stress response and pathogenesis.

EV-mediated transport of fungal RNA was recently shown in both commensal and opportunistic fungi. EV RNA molecules, mostly smaller than 250 nucleotides (nt), were identified in Cryptococcus neoformans, Paracoccidioides brasiliensis, Candida albicans, Saccharomyces cerevisiae, and Malassezia sympodialis (17, 18). Since H. capsulatum packages diverse compounds within EVs, we postulated that it too would use these compartments to export RNA. In this study, the EV-associated RNA components were characterized in two different isolates of H. capsulatum. As described in other fungi, H. capsulatum EVs carry both mRNAs and noncoding RNAs (ncRNAs). In addition, proteomic data allowed the identification of 139 RNA-binding proteins (RBPs) in the EVs, suggesting that proteins involved in RNA metabolism might play an important role in cell communication through the EVs. Our results add this important pathogen to the list of fungal species with the ability to use EVs for the extracellular release of RNA.

## RESULTS

### Histoplasma capsulatum EVs contain RNA.

We characterized the RNA molecules contained in EVs isolated from culture supernatant samples of H. capsulatum strains G186AR and G217B. These strains belong to distinct clades, and G217B has been shown to be more virulent than G186AR in experimental models (19, 20). The best-known difference between these two strains is that G217B lacks alpha-1,3-glucan on the yeast form cell wall (19, 20).

The reads obtained from the mRNA libraries (reads of >200 nt) were aligned with each strain-specific genome available at the NCBI (G186AR ABBS02 and G217B ABBT01). For data validation, we considered only sequences with expression values of transcripts per million (TPM) of ≥100 in all biological replicates and transcripts with reads covering at least 50% of the coding DNA sequence (CDS). The small RNA (sRNA) fraction was analyzed for the presence of different species of noncoding RNAs (ncRNAs) by aligning the sRNA fraction (reads of <200 nt) with the H. capsulatum G186AR strain. These RNA molecules were compared between the strains in order to gain insights into the role of the EV RNA in this fungus and also to determine if there were differences with respect to composition between the two strains with distinct phenotypes.

### Strain-specific content of EV RNA in H. capsulatum.

**FIG 1:**
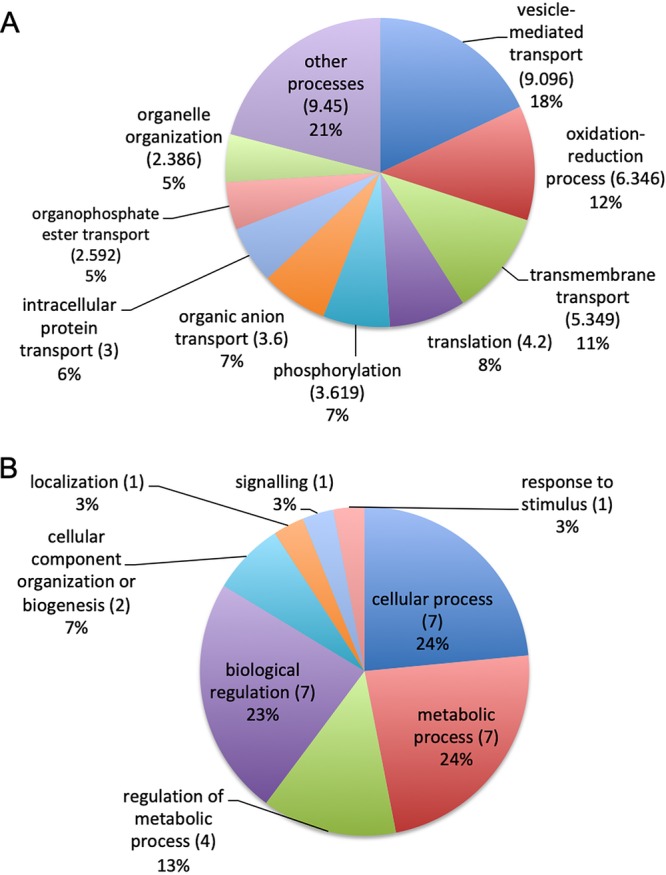
Gene ontology analysis. The pie charts present the gene ontology of mRNA sequences enriched in EVs isolated from (A) H. capsulatum G217B (*n* = 93) and (B) H. capsulatum G186AR (*n* = 31).

We identified a total of 124 mRNA sequences in EV samples from the two strains and carried out paired comparisons between the G186AR and G217B samples. We applied the statistical negative binomial test with filters corresponding to TPM values of ≥100, log2 values of ≥2, and false-discovery-rate (FDR) values of ≤0.05. We observed 93 transcripts enriched in EVs derived from the G217B strain, while 31 transcripts were enriched in the G186AR strain (see Table S1 in the supplemental material). In the G217B-associated transcripts, we observed enrichment in biological processes for vesicle-mediated transport (18%), oxidation-reduction mechanisms (12%), transmembrane transport (11%), and translation (8%) (Fig. 1). In the G186AR strain, the mRNA sequences were enriched only in general cellular and metabolic processes (59%). These results suggest that there are important differences with respect to the mRNA composition of EVs derived from these two strains of H. capsulatum.

10.1128/mSphere.00176-19.1 TABLE S1 List of transcripts differentially enriched in H. capsulatum G217B and G186AR strains. Download Table S1, XLSX file, 1.4 MB. Copyright © 2019 Alves et al. 2019 Alves et al. This content is distributed under the terms of the Creative Commons Attribution 4.0 International license.

### H. capsulatum EVs contain mRNA fragments and microRNA (miRNA)-like molecules.

In addition to the identification of full-length transcripts in EVs, we also detected short reads of averages of 25 to 40 nt in length that aligned consistently in the CDS but at specific positions of the mRNAs (3′ end, 5′ end, or middle sequence); about 50% of these short fragments aligned to the reverse strand, including 172 (G217B) and 80 (G186AR) sequences of this type (Table 1). A total of 172 fragments were represented in the G217B sample compared to only 80 in the G186AR EVs (Table 1). About 47% of the reference mRNA translate proteins of unknown biological processes; this could be explained by the fact that around 33% of the genes annotated in H. capsulatum genome code hypothetical proteins and/or do not present a conserved domain, which impedes our current ability to determine specific biological activities. Those associated with DNA metabolism/biogenesis were the second most abundant for both EV samples (22 for G217B versus 16 for G186AR), followed by transport for G217B and by protein modification for both strain EVs. Other processes related to short RNAs identified in both strain EVs were oxidation-reduction, signaling, and carbohydrate and lipid metabolism (Table 1). RNA fragments associated with translation were highly enriched in G217B (*n* = 11) but not in G186AR (*n* = 2) EVs, while those related to response to stress were found exclusively in the G217B sample. The corresponding proteins are stress response protein whi2, DNA repair protein rad5, and a thermotolerance protein (Table 1). Analysis of translation-related sequences allowed identification of mRNA fragments associated with distinct steps of the translation process, such as ribosome biogenesis and processing. Other metabolic pathways identified in both strains were protein modification, carbohydrate, and lipid metabolism, signaling, oxidation-reduction, and transmembrane transport, among others (Table 1).

**TABLE 1:** Fragments of mRNAs identified in the EVs isolated from the G217B and G186AR strains^*a*^

Feature ID	G217B alignment	G186AR alignment	Sequence description	GO
Protein modification				
HCBG_03026	5′R	5′R	Tetratricopeptide-like helical	Amino acid metabolic process
HCBG_05660	MR		CMGC SRPK protein kinase	Protein modification process
HCBG_05782	MF		Dihydrofolate synthetase fol3	Cofactor metabolic process
HCBG_06582	5′F		Aspartyl aminopeptidase	Peptidase activity
HCBG_07777	MF		Mitochondrial processing peptidase alpha	Peptidase activity
HCBG_08965	MF	MF	Tyrosine phosphatase	Protein modification process
HCBG_09127	3′R / 3′F		Proteasome component C5	Peptidase activity
HCBG_09175	5′F	5′F	Aspartic-type endopeptidase	Peptidase activity
HCBG_09182	MR		Protein kinase	Protein modification process
HCBG_01228	5′F		Oxidative stress-induced growth inhibitor 2	Peptidase activity
HCBG_01665	MF	MF	pH domain-containing protein	Protein modification process
HCBG_03811	MR	3′R	Heat shock protein Hsp98 Hsp104	ATPase activity, peptidase activity
HCBG_00544	MF		Ubiquitin conjugating enzyme	Ligase activity
HCBG_02715	3′F	3′F	Ubiquitin family protein	
HCBG_05116	3′F		Protein	Protein modification process
HCBG_07497		3′F	Protein	Peptidase activity

Carbohydrate metabolism				
HCBG_00058	5′R		Mannosyl-oligosaccharide alpha-mannosidase	Catabolic process
HCBG_00633	3′R / 3′NS		Class V chitinase	Catabolic process
HCBG_06620	3′R	3′R	Transaldolase	Carbohydrate metabolic process

Lipid metabolism				
HCBG_02433	MF	5′F	Acyl carrier protein	Biosynthetic process
HCBG_01540	MF	MF	Predicted protein	Lipid metabolic process
HCBG_04372		3′R	GPI anchor biosynthesis protein (Pig-f)	Lipid metabolic process

Response to stress				
HCBG_02224	3′F		General stress response protein Whi2	
HCBG_01643	3′R		DNA repair protein Rad5	Response to stress
HCBG_06196	3′R		Thermotolerance protein	

Translation				
HCBG_00808	MF	MF	60S ribosomal protein L15	
HCBG_00853	3′F		Small nucleolar ribonucleoprotein complex	
HCBG_01544	5′R / F	5′R	Ribosome biogenesis protein	
HCBG_02168	5′F / MF		60S ribosomal protein l25	Translation
HCBG_02499	5′R		rRNA processing protein Utp6	Oxidoreductase activity
HCBG_02762	3′F		60S ribosomal protein L31	Translation
HCBG_04580	MR		Prenyl cysteine carboxyl methyltransferase Ste14	mRNA processing
HCBG_08644	5′R		Leucyl-tRNA synthetase	Translation
HCBG_03984	5′R		Transcription initiation protein Spt5	Translation
HCBG_04793	5′R		U5 small nuclear ribonucleoprotein component	Chromosome organization
HCBG_06802	5′R		Ribosome biogenesis protein Ssf2	

Signaling process				
HCBG_00598	5′F / 5′NS		MinD kinetochore complex component Nnf1	Signal transduction
HCBG_03086*	5′R / F		Ste Ste20 paka protein kinase	Reproduction
HCBG_04646*		3′R	Protein Ras-2	Signal transduction

Oxidation-reduction				
HCBG_00763	3′R	3′R / 3′NS	Benzoate 4-monooxygenase cytochrome p450	Oxidoreductase activity
HCBG_03251	3′R / 3 F		Tim-barrel enzyme family protein	Oxidoreductase activity
HCBG_04436	5′R / 3′R		Flavin-containing monooxygenase	Oxidoreductase activity
HCBG_05481	3′F	3′F	Like subfamily b member 4	Protein folding
HCBG_05591	3′F	3′F	Fmn-binding split-barrel-like protein	Oxidoreductase activity
HCBG_06890	5′F		Glutaredoxin	Homeostatic process
HCBG_08366	3′F		Conserved hypothetical protein	Oxidoreductase activity
HCBG_01233	5′R / 5′F		Galactose oxidase beta-propeller	
HCBG_00232		5′F	Tyrosinase	Oxidoreductase activity
HCBG_03159		MR	Ste Ste7 Mek1 protein kinase	Reproduction

Transport				
HCBG_00485	3′R		Vacuolar ABC heavy-metal transporter	Transmembrane transport
HCBG_00680	3′F		Arsenine resistance protein	Transmembrane transport
HCBG_00850	MR		MFS monocarboxylate	Transmembrane transport
HCBG_01089	5′F / 5′NS	5′R / 5′NS	Mitochondrial carrier	Transport
HCBG_02374	5′R		Endosomal cargo receptor	Vesicle-mediated transport
HCBG_02985	5′R	5′R	V-type proton ATPase proteolipid subunit	Vesicle-mediated transport
HCBG_03067	5′R	5′R	Mitochondrial dicarboxylate carrier	Transmembrane transport
HCBG_03738		MF	Exocyst complex component Sec10	Vesicle-mediated transport
HCBG_04312	3′F	5′R / 3′F	Nonrepetitive nucleoporin	Nucleocytoplasmic transport
HCBG_04317	5′F		mRNA transport regulator	Transport
HCBG_04719	5′F		Nucleoporin	
HCBG_04608	3′R		MFS transporter	Transmembrane transport
HCBG_05671	MR		Actin-associated protein	Vesicle-mediated transport
HCBG_05941	5′F	5′R	Potassium uptake protein	Transmembrane transport
HCBG_05942	MR		Potassium uptake protein	Transmembrane transport
HCBG_06437	MF	MF	Oligopeptide transporter	Transport
HCBG_06658	MR		PX domain-containing protein	Transmembrane transport
HCBG_07112	MF		Ap-2 adaptor complex subunit	Vesicle-mediated transport
HCBG_07566	3′R	3′R / MR	Actin cytoskeleton-regulatory complex protein Pan1	Vesicle-mediated transport
HCBG_08252*	5′F		MFS multidrug transporter	Transmembrane transport
HCBG_09093	5′R		Kinetoplast-associated protein Kap	Transmembrane transport
HCBG_09150	5′R / 3′R		Cap binding protein	Transport
HCBG_04513	5′F		3-Oxoacyl-acyl-carrier-protein synthase	

DNA metabolism or biogenesis				
HCBG_00397		MF	PHD finger domain	Chromosome organization
HCBG_00799	5′F	5′F	Transcriptional regulator Ngg1	Peptidase activity
HCBG_01145	5′R	5′R / 3′F	C6 zinc finger domain-containing protein	Biosynthetic process
HCBG_02996	3′F		Recombination hot spot-binding protein	DNA metabolic process
HCBG_01721	3′F		Nitrogen assimilation transcription factor nira	Chromosome organization
HCBG_03125		MF	White collar	Signal transduction
HCBG_03879	MR	MR	DNA-directed RNA polymerase I subunit	Biosynthetic process
HCBG_04485		3′F	Centromere protein Cenp-o	Chromosome organization
HCBG_04625	MR		C6 finger domain	Biosynthetic process
HCBG_04221	3′R		Chromatin remodeling complex subunit	Helicase activity
HCBG_05411	3′R	3′R	Transcription factor SteA	Reproduction
HCBG_05417	MF		Elongator complex protein 3	Biosynthetic process
HCBG_05986	5′F		G_1/S_ regulator	DNA metabolic process
HCBG_05814	3′R	3′R	Histone H2a	Chromosome organization
HCBG_06244		MF	double-strand-break repair protein	DNA metabolic process, reproduction
HCBG_07395	MR		CP2 transcription factor	Biosynthetic process
HCBG_07428	3′F		Caf1 family ribonuclease	
HCBG_09164	MF	MF	C2H2 finger domain transcription factor	Biosynthetic process
HCBG_00846	5′F		Transcription factor Tau55-like protein	
HCBG_04340	3′R	3′R	Formamidopyrimidine-DNA glycosylase	DNA metabolic process
HCBG_01534	MF	MF	Telomere length regulation protein Elg1	Ion binding, lipid binding
HCBG_06146	5′R	5′R	Telomerase-binding protein Est1a	
HCBG_07560	5′R / 5′F	5′R / 5′F	DNA repair protein protein	
HCBG_05625	3′R	3′R	p60-like cell wall	
HCBG_09024	MR		Hlh transcription factor	
HCBG_06915	5′F	5′F	Proline-rich protein-15	Chromosome segregation

Other/unknown function				
HCBG_00048	5′R	5′R	Hypothetical protein HCBG_00048	
HCBG_00453	5′R		MIZ zinc finger protein	Ion binding
HCBG_00947	3′F		Predicted protein	
HCBG_00975	5′R	5′R	ATPase AAA-5 protein	Ion binding
HCBG_01015	MF	MF	Predicted protein	
HCBG_01082	3′R / 3′F	3′R	Zinc knuckle domain protein	
HCBG_01086	5′R		Predicted protein	
HCBG_01127	5′R / 3′R		Predicted protein	
HCBG_01146	MF		Predicted protein	
HCBG_01161	MF		Predicted protein	
HCBG_01256	3′R		Conserved hypothetical protein	
HCBG_01258	MR		Predicted protein	
HCBG_01500	MR		Predicted protein	
HCBG_01656	MF		Predicted protein	
HCBG_01888	3′R	3′R	Conserved hypothetical protein	
HCBG_01952	3′F		Conserved hypothetical protein	
HCBG_02098	5′R		Protein	
HCBG_02107	5′F		Predicted protein	
HCBG_02158		3′F	Conserved hypothetical protein	
HCBG_02464	3′R / 3′F	3′F / 3′R / 3′NS	Carbohydrate-binding module family 48 protein	
HCBG_02569	MR / MF	MF	Predicted protein	
HCBG_02659	MR / MF	MR	Predicted protein	
HCBG_02697	3′R	3′R	Predicted protein	
HCBG_02981	MF		Phosphotransferase enzyme family protein	
HCBG_02986	MF	5′F	Predicted protein	
HCBG_03093	MR		PH domain protein	
HCBG_03374	MF	MF	Glutathione transferase	
HCBG_03658	3′R / 3F		Conserved hypothetical protein	Helicase activity
HCBG_03692	3′R / 3F		Predicted protein	
HCBG_03693	MR / MF	MR / MF	Predicted protein	
HCBG_03805	MF	MF	mtDNA inheritance protein	
HCBG_03899	MR	MR / 3′R	WD repeat protein	
HCBG_03911	3′R	3′R	Protein	
HCBG_03913	MR		Hypothetical protein HCBG_03913	
HCBG_03980	MR		Phosphatidylserine decarboxylase	
HCBG_04009	MR		Hypothetical protein HCBG_04009	
HCBG_04186	MR		Conserved hypothetical protein	
HCBG_04193	3′R	3′R	Conserved hypothetical protein	
HCBG_04201	3′F		Hypothetical protein HCBG_04201	
HCBG_04208	3′F	3′F	Conserved hypothetical protein	
HCBG_04365	MF		Hypothetical protein HCBG_04365	
HCBG_04371	5′R / 5′F		Bifunctional uridylyltransferase uridylyl-removing enzyme	
HCBG_04380	3′R	3′R	Predicted protein	
HCBG_04393	3′R		Protein	
HCBG_04452	3′R	3′R	Predicted protein	
HCBG_04780	5′R	5′R	Bromodomain-containing protein	
HCBG_04887		MR	Predicted protein	
HCBG_05336	5′R		UPF0160 domain protein	
HCBG_05404	3′R / 3′F		Predicted protein	
HCBG_05580	3′R		Methyltransferase domain-containing protein	
HCBG_05638	5′R		Predicted protein	
HCBG_05703	5′R		Conserved hypothetical protein	
HCBG_05744	5′F		T-complex protein 1 subunit beta	
HCBG_05763	3′R	3′F	Conserved hypothetical protein	
HCBG_05878	3′F		Hypothetical protein HCBG_05878	
HCBG_06018	5′F		Cytomegalovirus GH-receptor family	
HCBG_06054	MR		Phosphotransferase family protein	Ion binding, kinase activity
HCBG_06071	MF	MF	Protein	
HCBG_06082	MR		Conserved hypothetical protein	
HCBG_06114	3′F		Protein	
HCBG_06176	3′F		KH domain protein	RNA binding
HCBG_06239		5′R	Nonsense-mediated mRNA decay protein	
HCBG_06270	MR		Predicted protein	
HCBG_06364	MR		F-box domain-containing protein	
HCBG_06436	MF		Predicted protein	
HCBG_06661		5′NS	Predicted protein	
HCBG_06677	3′F		Predicted protein	
HCBG_06927	3′R / 3′F		Predicted protein	
HCBG_07002	5′R / 5′F	5′R / 5′F	Ketoreductase	
HCBG_07065	5′F		Predicted protein	
HCBG_07214	5′R	5′R	Predicted protein	
HCBG_07247	MR		Acyltransferase 3	Transferring acyl groups
HCBG_07296	MR	MR	Hypothetical protein HCBG_07296	
HCBG_07377	MF	MR	Predicted protein	
HCBG_07484	3′F		Rhomboid family membrane protein	Peptidase activity
HCBG_07611	MR / MF	MR / MF / MNS	Protein	
HCBG_07676	3′R / 3′F		Lyr family protein	
HCBG_07802	3′R / 3′F	3′R / 3′F	Predicted protein	
HCBG_07811	3′F	3′F	Predicted protein	
HCBG_08059	MR	MF	DUF833 domain protein	Protein complex assembly
HCBG_08505	3′F		Sucrase ferredoxin domain-containing protein	
HCBG_08661	MF	MF	Predicted protein	
HCBG_08693	3′R		Set domain protein	
HCBG_08838	5′R		WW domain	
HCBG_08850	5′R		Integral membrane protein	
HCBG_09013	5′F	5′F	Predicted protein	
HCBG_09099	5′R	5′R	Conserved hypothetical protein	
HCBG_09144	MF		Predicted protein	

a For some transcripts, there was an alignment in specific positions of the mRNA, not covering the entire sequence. 5′, 3′, or M (middle of the mRNA) followed by an “F” or an “R” represents forward (F) or reverse (R) orientation. GO, gene ontology; GPI, glycosylphosphatidylinositol; ID, identifier; mtDNA, mitochondrial DNA.

**FIG 2:**
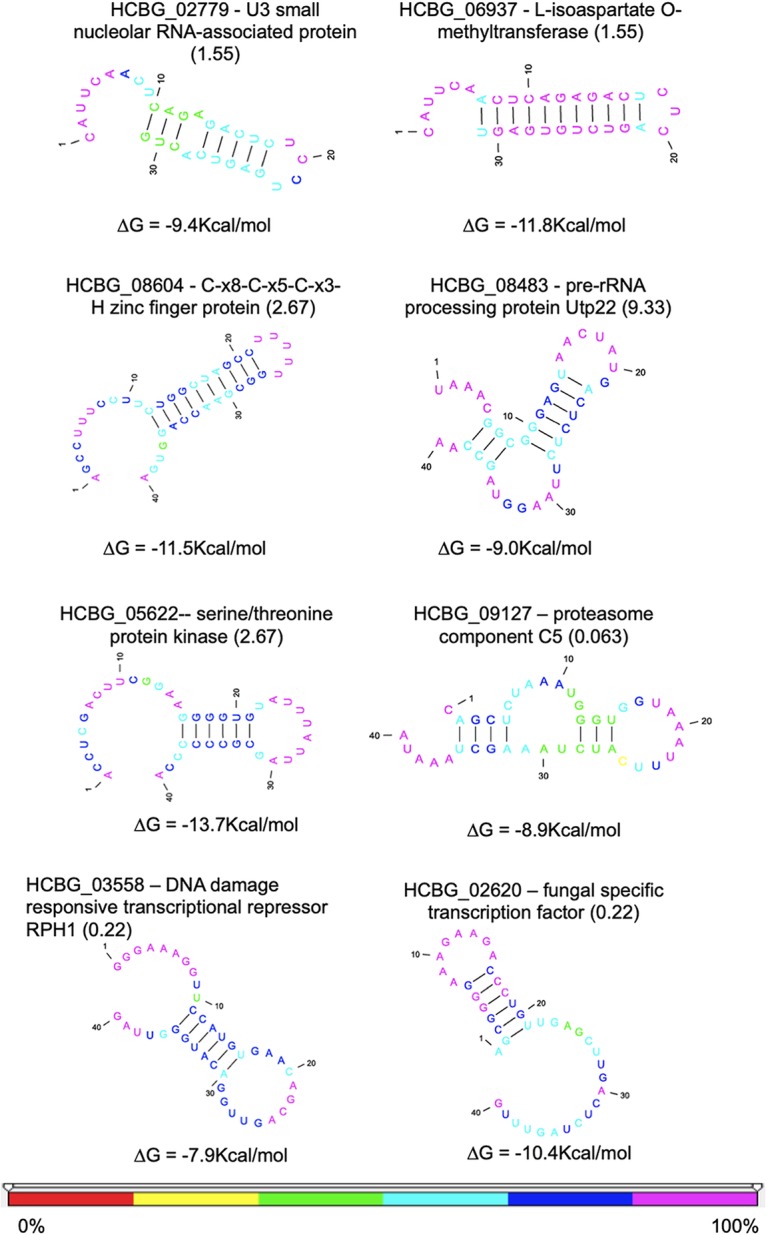
RNA secondary structure. We used ppFold software to predict the secondary structure from the putative miRNAs extracted from the obtained reads. The numbers in parentheses represent the alignment E values. The colors indicated for the nucleotides represent the reliability percentage for each position of the RNA molecule (bottom panel). The stability value corresponding to each structure is given in kilocalories/mole.

To gain further insight into the role of EV RNAs, to determine if they could be derived from a miRNA-like pathway, and to assess if they could play a biological role in the recipient cell, we searched for RNA secondary structures, since they are fundamental for gene expression regulation (21). A broad study of RNA structures in distinct cells revealed regulatory effects of the RNA structure throughout mRNA life cycle such as polyadenylation, splicing, translation, and turnover (22, 23). Using the entire range of EV RNA sequencing (RNA-seq) data, a total of 33 RNAs with putative structures were generated by a probability distribution, using a free energy (ΔG) value of less than or equal to −7.0 (Table S2). On the basis of this parameter, we identified transcripts for U3 small nucleolar RNA-associated protein, l-isoaspartate O-methyltransferase, serine/threonine-protein kinase, proteasome component C5, pre-rRNA processing protein Utp22, C-x8-C-x5-C-x3-H zinc finger protein, fungus-specific transcription factor domain-containing protein, and DNA damage-responsive transcriptional repressor RPH1 (Fig. 2; see also Table S2).

10.1128/mSphere.00176-19.2 TABLE S2 Comparison of the RNAs with predicted secondary structure with the H. capsulatum genome. Download Table S2, XLSX file, 0.01 MB. Copyright © 2019 Alves et al. 2019 Alves et al. This content is distributed under the terms of the Creative Commons Attribution 4.0 International license.

### Comparison of EV ncRNA classes in H. capsulatum EVs.

We used the ncRNA database from H. capsulatum to identify the classes of ncRNA present in EV RNAs. The data analysis revealed 73 different sequences of ncRNA in H. capsulatum EVs from the G186AR strain and 38 from the G217B isolate. A total of 33 molecular species were common to both strains, 40 were exclusively identified in the G186AR strain, and the most abundant class of ncRNA found in H. capsulatum EVs consisted of tRNAs (Table 2).

**TABLE 2:** Classes of ncRNA sequences identified in EV preparations from *H. capsulatum* strains G186AR and G217B^*a*^

RNA category and ncRNA	G186AR	G217B
rRNA		
15S_rRNA	—	X
NTS1-2	X	—
RDN18-1	X	X
RDN18-2	X	X
RDN25-1	X	—
RDN25-2	X	X
RDN37-1	X	—
RDN37-2	X	—
RDN5-1	X	X
RDN5-2	X	X
RDN5-3	X	X
RDN5-4	X	X
RDN5-5	X	X
RDN5-6	X	X
RDN58-1	X	X
RDN58-2	X	X

ncRNA		
RUF21	X	X

snoRNA		
snR54	X	X

tRNA		
tRNA-Ser	—	X
tRNA-Met	—	X
tRNA-Gln	—	X
tRNA-Cys	—	X
tRNA-Ser	X	X
tRNA-Pro	X	X
tRNA-Ala	X	X
tRNA-Thr	X	X
tRNA-Ala	X	X
tRNA-Phe	X	X
tRNA-Ala	X	X
tRNA-Asn	X	X
tRNA-Met	X	X
tRNA-Arg	X	X
tRNA-Trp	X	X
tRNA-Gly	X	X
tRNA-Asp	X	X
tRNA-Pro	X	X
tRNA-Thr	X	X
tRNA-His	X	X
tRNA-Glu	X	X
tRNA-Gln	X	X
tRNA-Tyr	X	X
tRNA-Gln	X	X
tRNA-Gly	X	—
tRNA-Lys	X	—
tRNA-Ile	X	—
tRNA-Leu	X	—
tRNA-Met	X	—
tRNA-Gly	X	—
tRNA-Ile	X	—
tRNA-Thr	X	—
tRNA-Lys	X	—
tRNA-Met	X	—
tRNA-Val	X	—
tRNA-Phe	X	—
tRNA-Ile	X	—
tRNA-Sec	X	—
tRNA-Asp	X	—
tRNA-Thr	X	—
tRNA-Ile	X	—
tRNA-Ser	X	—
tRNA-Ser	X	—
tRNA-Arg	X	—
tRNA-Lys	X	—
tRNA-Leu	X	—
tRNA-Ser	X	—
tRNA-Leu	X	—
tRNA-Ala	X	—
tRNA-Cys	X	—
tRNA-Thr	X	—
tRNA-His	X	—
tRNA-Tyr	X	—
tRNA-Ser	X	—
tRNA-Leu	X	—
tRNA-Lys	X	—
tRNA-Ala	X	—
tRNA-Pro	X	—
tRNA-Arg	X	—
tRNA-Glu	X	—

a X, present; —, absent.

### Analysis of proteins putatively associated with RNA metabolism in the EVs.

As a rule, cellular RNAs are covered with proteins and exist as ribonucleoprotein (RNP) complexes. The proteins associated with RNAs are named RNA-binding proteins (RBPs). These proteins participate in several biological processes, ranging from transcription to RNA decay (24). In this context, we investigated the presence of RBPs in the H. capsulatum EVs. We analyzed the proteomic EV data available for the G217B strain (25), and we identified 139 proteins related to RNA metabolism (8) (Table 3; see also Table S3). We found many RBPs, such as poly(A) binding protein (PABP), Nrd1, Prp24, and Snd1; splicing factors, exosome complex components, and ribosomal proteins (Table 3; see also Table S3) were identified. In addition, we also found quelling-deficient protein 2 (QDE2), an Argonaute protein important in the RNA machinery in fungi. Because we identified the QDE2 in EVs, we searched for the components of the RNA interference (RNAi) machinery in H. capsulatum and compared them with the proteins from Neurospora crassa and Schizosaccharomyces pombe, which are the fungal species for which the RNAi machinery was best described previously (26, 27). H. capsulatum EVs contained one Argonaute protein (QDE2), two Dicer-like proteins, the QIP (quelling interaction protein), and the RNA-dependent RNA polymerase (QDE1) (Table 4).

**TABLE 3:** Proteins related to RNA metabolism identified in EV preparations from *H. capsulatum* strain G217B

Majority protein ID	Protein name	Gene name
C0NMG7	QDE2 protein	HCBG_03944
C0P170	Cap binding protein	HCBG_09150
C0NJ23	Exosome complex exonuclease RRP4	HCBG_03153
C0NM03	Exosome complex exonuclease RRP45	HCBG_04533
C0NCT3	KH domain RNA-binding protein	HCBG_00929
C0NUH0	KH domain RNA-binding protein	HCBG_07001
C0NIU5	KH domain-containing protein	HCBG_02352
C0NUS5	mRNA 3′-end-processing protein RNA14	HCBG_06689
C0NNW0	mRNA cleavage and polyadenylation factor CLP1	CLP1 HCBG_04840
C0NP91	mRNA decapping enzyme	HCBG_04971
C0NC87	mRNA export factor Mex67	HCBG_00733
C0NJ33	Nuclear and cytoplasmic polyadenylated RNA-binding protein Pub1	HCBG_03163
C0NQQ9	Poly(A)^+^ RNA export protein	HCBG_05339
C0NSS5	Polyadenylate-binding protein (PABP)	HCBG_06205
C0NKR4	Ribonucleoprotein	HCBG_03744
C0NSY4	RNA binding domain-containing protein	HCBG_06264
C0NWH9	RNA-binding protein	HCBG_07509
C0NB22	RNA-binding protein	HCBG_00318
C0NPA1	RNA-binding protein Nrd1	HCBG_04981
C0NZI9	RNA-binding protein Prp24	HCBG_08569
C0NTZ5	RNA-binding protein Snd1	HCBG_06625
C0NMQ0	RNP domain-containing protein	HCBG_04027
C0NLQ4	RRM domain-containing protein	HCBG_04434
C0NJ27	Transcription elongation factor Spt6	HCBG_03157
C0NTQ1	Transcription initiation factor TFIID complex 60-kDa subunit	HCBG_06531
C0NRU6	U1 snRNP-associated protein Usp106	HCBG_05876
C0NZZ2	U1 snRNP-associated protein Usp107	HCBG_08722
C0NBS3	U2 snRNP auxiliary factor large subunit	HCBG_00569
C0NAD4	U3 small nucleolar RNA-associated protein	HCBG_00080
C0NZA3	U3 small nucleolar RNA-associated protein 22	HCBG_08483
C0NLW4	U3 snoRNP-associated protein Rrp5	HCBG_04494
C0P0R0	U6 snRNA-associated Sm-like protein LSm2	HCBG_08990
C0P041	30S ribosomal protein S10	HCBG_08883
C0NFV8	40S ribosomal protein S15	HCBG_01774
C0NX47	40S ribosomal protein S18	HCBG_08039
C0NZD2	40S ribosomal protein S20	HCBG_08512
C0NBD0	40S ribosomal protein S21	HCBG_00426
C0NUD0	40S ribosomal protein S3	HCBG_06961
C0NLP3	40S ribosomal protein S4	HCBG_04423
C0NF40	40S ribosomal protein S5A	HCBG_01506
C0NLR5	40S ribosomal protein S9	HCBG_04445
C0NTH6	5′–3′ exoribonuclease 1 (EC 3.1.13.-)	HCBG_06456
C0NKI2	60S ribosomal protein L1	HCBG_03662
C0NNL2	60S ribosomal protein L3	HCBG_04742
C0NCP3	60S ribosomal protein L30	HCBG_00889
C0NRD6	60S ribosomal protein L5	HCBG_05566
C0NQR6	60S ribosomal protein L9B	HCBG_05346
C0NPC0	Acyl-RNA-complex subunit	HCBG_05000
C0NKL8	Alanine-tRNA ligase (EC 6.1.1.7) (alanyl-tRNA synthetase) (AlaRS)	ALA1 HCBG_03698
C0NCS0	Alternative oxidase (EC 1.-.-.-)	HCBG_00916
C0ND66	Arginyl-tRNA synthetase	HCBG_01062
C0NT82	Asparagine-rich protein	HCBG_06362
C0NP94	Asparaginyl-tRNA synthetase	HCBG_04974
C0NGY7	Aspartyl-tRNA synthetase	HCBG_02609
C0NNJ3	ATP-dependent helicase NAM7	HCBG_04723
C0NIT7	ATP-dependent RNA helicase DOB1	HCBG_02344
C0NAN2	ATP-dependent RNA helicase EIF4A	HCBG_00178
C0NFC7	Cell cycle control protein	HCBG_01593
C0NT49	Cleavage and polyadenylation specific factor 5	HCBG_06329
C0NW18	Clustered mitochondria protein homolog (protein TIF31 homolog)	CLU1 TIF31 HCBG_07348
C0NTW5	Cysteinyl-tRNA synthetase	HCBG_06595
C0NZE4	d-Aminoacyl-tRNA deacylase (EC 3.1.1.-) (EC 3.1.1.96)	HCBG_08524
C0NSH0	DNA-directed RNA polymerase II polypeptide	HCBG_06100
C0NB61	DNA-directed RNA polymerase subunit beta (EC 2.7.7.6)	HCBG_00357
C0NKS3	Elicitor protein	HCBG_03753
C0NRY6	Eukaryotic peptide chain release factor GTP-binding subunit	HCBG_05916
C0P0 × 7	Eukaryotic translation initiation factor 3 subunit D (EIF3D)	HCBG_09057
C0NEV9	Fibrillarin	HCBG_01425
C0NZT8	Glutaminyl-tRNA synthetase	HCBG_08668
C0NKS5	Glutamyl-tRNA synthetase	HCBG_03755
C0NE28	Glycyl-tRNA synthetase	HCBG_02121
C0NN35	Histidyl-tRNA synthetase	HCBG_04162
C0NL66	Isoleucyl-tRNA synthetase, cytoplasmic	HCBG_03896
C0NZR4	Leucyl-tRNA synthetase	HCBG_08644
C0NH95	Leucyl-tRNA synthetase	HCBG_02717
C0NI62	Lysine-tRNA ligase (EC 6.1.1.6) (lysyl-tRNA synthetase)	HCBG_03034
C0NMS8	Mitotic control protein dis3	HCBG_04055
C0NBJ8	mRNA splicing protein PRP8	HCBG_00494
C0NY83	NAM9^+^ protein	HCBG_07877
C0NG69	Nucleic acid-binding protein	HCBG_01885
C0NUD1	Phenylalanyl-tRNA synthetase subunit beta	HCBG_06962
C0NBD1	Phenylalanyl-tRNA synthetase subunit beta cytoplasmic	HCBG_00427
C0NUP1	Polymerase II polypeptide D	HCBG_06655
C0NNC4	Pre-mRNA-processing factor 39	HCBG_04251
C0NJB4	Pre-mRNA-processing protein prp40	HCBG_03244
C0NXM8	Pre-mRNA-splicing factor	HCBG_08220
C0NLW7	Prolyl-tRNA synthetase	HCBG_04497
C0NW72	Ribonuclease T2-like protein	HCBG_07402
C0NEF9	Ribonuclease Z	HCBG_01275
C0NIJ3	Ribosomal biogenesis protein Gar2	HCBG_02250
C0NHN4	Ribosomal protein L14	HCBG_02856
C0NI43	Ribosomal protein L6	HCBG_03015
C0NVX9	Ribosomal protein S5	HCBG_07309
C0NN82	RNA helicase (EC 3.6.4.13)	HCBG_04209
C0NEY2	RNA polymerase II largest subunit	HCBG_01448
C0NL28	RNA polymerase subunit	HCBG_03858
C0NYA7	RNase H domain-containing protein	HCBG_07901
C0NH14	RNP domain-containing protein	HCBG_02636
C0NDP9	RNP domain-containing protein	HCBG_01992
C0NC99	SAM domain-containing protein	HCBG_00745
C0NE91	Seryl-tRNA synthetase	HCBG_02184
C0NSR2	Signal recognition particle subunit SRP68 (SRP68)	HCBG_06192
C0NDB1	Small nuclear ribonucleoprotein	HCBG_01107
C0NTA0	Splicing factor 3A subunit 3	HCBG_06380
C0NUB9	Splicing factor 3B	HCBG_06950
C0NBR2	Splicing factor 3B subunit 1	HCBG_00558
C0NGZ9	Threonyl-tRNA synthetase	HCBG_02621
C0NSB0	Transfer RNA-Trp synthetase	HCBG_06040
C0NL23	tRNA (cytosine-5-)-methyltransferase NCL1	HCBG_03853
C0NUP2	tRNA [guanine(37)-N1]-methyltransferase (EC 2.1.1.228)	TRM5 HCBG_06656
C0NEY0	tRNA guanylyltransferase	HCBG_01446
C0NJJ2	tRNA ligase (EC 6.5.1.3)	HCBG_03322
C0NM44	tRNA pseudouridine synthase	HCBG_04574
C0NSG9	Tyrosine-tRNA ligase (EC 6.1.1.1) (Tyrosyl-tRNA synthetase)	HCBG_06099
C0NP46	Uncharacterized protein	HCBG_04926
C0NZF6	Uncharacterized protein	HCBG_08536
C0NIA9	Uncharacterized protein	HCBG_03081
C0NMF3	Uncharacterized protein	HCBG_04683
C0NPI9	Uncharacterized protein	HCBG_05069
C0NKI6	Uncharacterized protein	HCBG_03666
C0NF97	Uncharacterized protein	HCBG_01563
C0NEJ1	Uncharacterized protein	HCBG_01307
C0NEC3	Uncharacterized protein	HCBG_01239
C0NJN9	Uncharacterized protein	HCBG_03369
C0NYC3	Uncharacterized protein	HCBG_07917
C0NIB5	Uncharacterized protein	HCBG_03087
C0NYN4	Uncharacterized protein	HCBG_08264
C0NBT4	Uncharacterized protein	HCBG_00580
C0NKE4	Uncharacterized protein	HCBG_03624
C0NGB7	Uncharacterized protein	HCBG_02389
C0NM01	Uncharacterized protein	HCBG_04531
C0NG47	Uncharacterized protein	HCBG_01863
C0NEU7	Uncharacterized protein	HCBG_01413
C0NG27	Valyl-tRNA synthetase	HCBG_01843
C0P019	Vip1 protein	HCBG_08749
C0NG23	Ribosome biogenesis protein RPF2	HCBG_01839
C0NGE8	Ribosome biogenesis protein TSR3	TSR3 HCBG_02420
C0NAE4	Ribosome biogenesis protein YTM1	YTM1 HCBG_00090

**TABLE 4:** Proteins associated with the RNAi machinery in *H. capsulatum* G186AR EVs compared to *S. pombe* and *N. crassa*

Protein	H. capsulatum product	G186AR ID	E value	% identity	% positives
NP_587782.1, argonaute (Schizosaccharomyces pombe)	QDE2 protein	HCBG_03944	1.00E−85	28	45
ESA42122.1, posttranscriptional silencing protein QDE-2 (Neurospora crassa OR74A)	QDE2 protein	HCBG_03944	1.00E−178	37	53
NP_588215.2, dicer (Schizosaccharomyces pombe)	Dicer-like protein	HCBG_01751	1.00E−113	28	44
EAA34302.3, dicer-like protein 2 (Neurospora crassa OR74A)	Dicer-like protein 2	HCBG_01136	3.00E−97	31	49
XP_959047.1, RNA-dependent RNA polymerase (Neurospora crassa OR74A)	RNA-dependent RNA polimerase	HCBG_06604	3.00E−92	31	46
XP_964030.3, RecQ family helicase (Neurospora crassa OR74A)	Dicer-like protein	HCBG_01751	0.00E + 00	45	60
ABQ45366.1, QDE-2-interacting protein (Neurospora crassa)	QDE-2-interacting protein (QIP)	HCBG_07373	2.00E−50	27	43

10.1128/mSphere.00176-19.3 TABLE S3 Proteins related to RNA metabolism identified in EVs derived from the H. capsulatum G217B strain (25). Download Table S3, XLSX file, 0.06 MB. Copyright © 2019 Alves et al. 2019 Alves et al. This content is distributed under the terms of the Creative Commons Attribution 4.0 International license.

### Comparisons of cellular RNA versus EV RNA showed a distinct enrichment of molecules in the vesicles.

**FIG 3:**
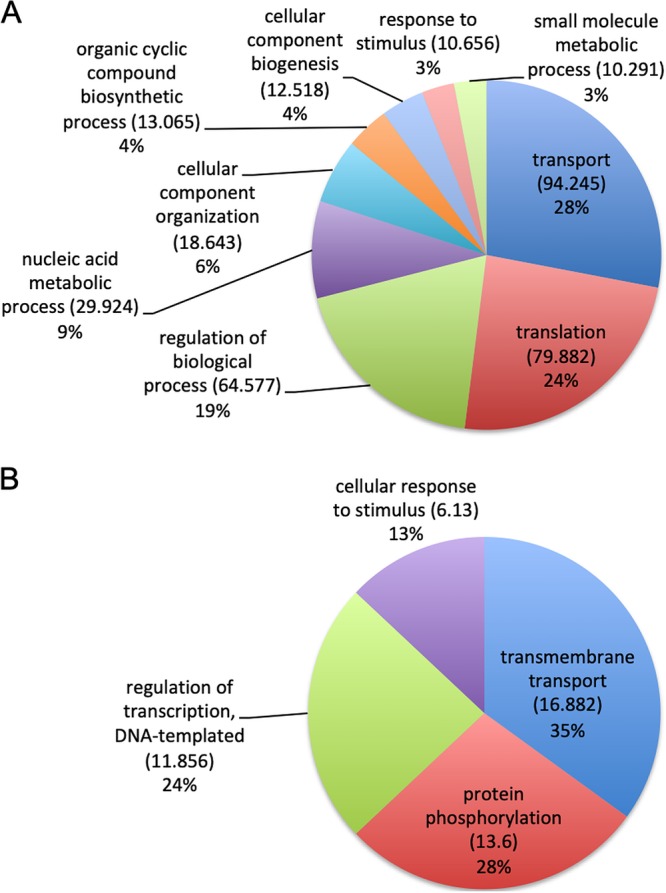
Gene ontology analysis. The pie charts present the gene ontology of mRNA sequences enriched in H. capsulatum cells (A) and in EVs isolated from H. capsulatum (B).

We next assessed the composition of cellular RNA from H. capsulatum yeast cells (28) and compared this information to that obtained from analyses of EV-associated RNA composition under the same conditions. There was no correlation between the transcripts with highest expression levels and their presence in the EVs (Table S4). Examples of highly expressed cellular transcripts included histones 4, 2B, and 2A, allergen Aspf4, chaperones, and translation factors, among others (Table S4). In contrast, zinc knuckle domain-containing protein, vacuolar ATP synthase subunit C, G_1/S_ regulator, thermotolerance protein, histone variant H2A.Z, and proteasome component C5 had an enrichment value of greater than 7,000 in the EVs, while they showed low expression values in the cell (Table S4). The differences in composition between cells and EVs were also evaluated by grouping the transcripts into biological processes (Fig. 3). For the yeast cells, the main pathways were associated with transport, translation, and general metabolic processes (Fig. 3). For the EVs, the enriched pathways were transmembrane transport, protein phosphorylation, and transcription regulation (Fig. 3). This result demonstrates the low levels of correlation between the most highly expressed cellular mRNAs and EV cargo, providing evidence that there might be a mechanism directing the RNA molecules to the EVs.

10.1128/mSphere.00176-19.4 TABLE S4 Comparison the H. capsulatum transcriptome (G186AR and G217B strains) (28) with the vesicular RNA sequences. Download Table S4, XLSX file, 2.2 MB. Copyright © 2019 Alves et al. 2019 Alves et al. This content is distributed under the terms of the Creative Commons Attribution 4.0 International license.

## DISCUSSION

As previously described (17, 18), RNA molecules associated with fungal EVs are remarkably diverse. For instance, mRNAs, tRNA fragments, snoRNAs, small nucleolar RNAs (snRNAs), and miRNA-like molecules were characterized in EVs from C. albicans, C. neoformans, P. brasiliensis, and S. cerevisiae (17). We observed similar distributions of RNA molecules in H. capsulatum EVs. The comparison between the G186AR and G217B EVs revealed important differences in the variety of mRNAs identified. When the mRNA composition was compared to what was described for other fungi, important similarities were observed. For example, the most abundant biological process identified in G217B EVs was vesicle-mediated transport, which was also the most abundant process in C. albicans EVs (17). Molecules required for ribosome biogenesis, which were observed in G217B EVs, belonged to the most highly enriched process in S. cerevisiae EVs (17). However, in the comparisons of the ncRNA molecules, different profiles were observed. Most of the ncRNAs in H. capsulatum strains derived from tRNAs; a similar profile was obtained with C. albicans (17). In addition, almost no snoRNAs were identified in H. capsulatum, but this class of ncRNAs was one of the most abundant in the EVs of other fungi (17). Differences in EV composition were observed previously in C. neoformans; the EV-associated RNA produced by mutant cells with defective unconventional secretion differed considerably from similar samples produced by wild-type cells (29).

In our study, we identified short reads that aligned specifically to exons; however, these sequences did not correspond to complete mRNAs in the EVs. They instead corresponded to 25-nt-long fragments that were enriched in specific exons of the transcript. These fragments of mRNAs were previously described in human cells (30), where most of the transcripts identified in the EVs corresponded to a fraction of the mRNA with an enrichment of the 3′ UTR of the transcript (30). The results of that human study led to the hypothesis that the mRNA fragments had a role in gene expression regulation in the recipient cells as the secreted mRNA could act as competitors to regulate stability, localization, and translation of mRNAs in target cells (30). In Mucor circinelloides cells, the presence of the RNA silencing pathway (sRNA) resulted in the production of both sense and antisense sRNAs (31–33). Sequencing analysis of the sRNA content of this fungus showed the existence of exonic small interfering RNAs (exo-siRNAs) as a new type of sRNA. They were produced from exons of the same genes that are later regulated through the repression of the corresponding mRNA (34). This result agrees with our observation of short reads in the exonic regions of the transcripts. We therefore hypothesize that, similarly to what was described for M. circinelloides cells, H. capsulatum EV fragments can regulate expression of their own mRNAs. Of note, we also found a highly represented population of putative exonic RNA in *Paracoccidioides* strains (R. Peres da Silva, L. V. G. Longo, J. P. C. da Cunha, T. J. P. Sobreira, H. Faoro, M. L. Rodrigues, S. Goldenberg, L. R. Alves, and R. Puccia, unpublished data).

As H. capsulatum EVs contain different RNA molecules, it is reasonable to hypothesize that proteins that regulate RNA metabolism are also present in the EVs, probably associated with RNA. If validated, this hypothesis could indicate how the RNAs in a specific subset are directed to the vesicles and exported. RNA-binding proteins (RBPs) participate in several biological processes, from RNA transcription to decay (24). We detected a number of RNA-binding proteins in H. capsulatum EVs (25). These proteins were also identified in association with EVs in other systems. For example, in the EVs produced by human epithelial cells, 30 RBPs were identified (35), including heterogeneous nuclear ribonucleoproteins (hnRNPs). These proteins are responsible for directing pre-mRNAs in the maturation processes that culminate in transcriptional regulation, alternative splicing, transport, and localization (35). In addition, RBPs in EVs were identified in distinct models as hepatocytes, human embryonic kidney (HEK) cells, and mouse myoblast cells (35–37). Interestingly, one of the RBPs identified in EVs was SND1 (staphylococcal nuclease domain-containing protein 1), which is a main component of the RNA-induced silencing complex (RISC) that plays an important role in miRNA function (37).

Another example of a protein identified in the EVs of H. capsulatum and distinct organisms is an endonuclease of the Ago2 family. An infection model with Plasmodium falciparum demonstrated that infected red blood cells released EVs containing functional miRNA-Argonaute 2 complexes (38). Moreover, endothelial cells internalized the P. falciparum EVs, and the miRNA-Argonaute 2 complexes were transferred to the cells and acted in regulation of gene expression and in the barrier properties of the recipient cells (38). The Argonaute protein named QDE2 in H. capsulatum was identified as enriched in the EVs of the G217B strain.

The small silencing RNAs include a variety of molecules, such as microRNAs (miRNAs) and various small interfering RNAs (siRNAs), including exo-siRNAs, endogenous siRNAs (endo-siRNAs), and Piwi-interacting RNAs (piRNAs) (39). Previous studies of small RNAs in fungi identified the RNAi machinery in the fission yeast species Schizosaccharomyces pombe, in the budding yeast species Saccharomyces castellii and C. albicans, and in filamentous fungi (26, 27, 40). One of the best-characterized models is represented by the filamentous fungus N. crassa (27, 41–45). The RNAi machinery in that organism functions in defense against transposons (46). A similar process has been described in C. neoformans, where RNAi is involved in the regulation of transposon activity and genome integrity during vegetative growth (47). In N. crassa, the *QDE2* gene encodes an Argonaute protein that is homologous to the rde-1 gene in C. elegans, encoding a protein required for double-stranded RNA (dsRNA)-induced silencing (27). The characterization of RNAs associated with QDE2 in N. crassa led to the identification of miRNA-like RNAs (milRNAs) in this organism (48). The identification of QDE2 in H. capsulatum EVs in association with the small RNAs indicated that the QDE2-milRNA complex might be directed to the EVs and possibly delivered to recipient cells, with the potential to interfere with gene expression regulation and/or cell-cell communication.

Fungal EVs have been implicated in a number of communication processes, including transfer of virulence (49) and antifungal resistance (50). In Cryptococcus gattii, pathogen-to-pathogen communication via EVs resulted in reversion of an avirulent phenotype through mechanisms that required vesicular RNA (49). The sequences required for this process, however, remained unknown. This is an efficient illustration of the potential derived from the characterization of EV-associated RNA in fungi. In this context, our study results provide information from the H. capsulatum model that will allow the design of pathogenic experimental models aiming at characterizing the role of extracellular RNAs in fungal pathogenesis.

## MATERIALS AND METHODS

### Fungal strains and growth conditions.

The H. capsulatum strains were subjected to long-term storage at −80°C. Aliquots were inoculated into Ham’s F-12 media (Gibco; catalog no. 21700-075) supplemented with glucose (18.2 g/liter), l-cysteine (8.4 mg/liter), HEPES (6 g/liter), and glutamic acid (1 g/liter) and cultivated at 37°C with constant shaking at 150 rpm. Viability assessments were performed using Janus green 0.02%, and all aliquots used had >99% live yeast cells. EVs were then isolated from fungal culture supernatants as previously described (12).

### sRNA isolation.

Small RNA-enriched fractions were isolated using a miRNeasy minikit (Qiagen) and were then treated with an RNeasy MinElute cleanup kit (Qiagen), according to the manufacturer’s protocol, to obtain small RNA-enriched fractions. The sRNA profile was assessed in an Agilent 2100 Bioanalyzer (Agilent Technologies).

### RNA sequencing.

Purified sRNA (100 ng) was used for RNA-seq analysis with two independent biological replicates. The RNA-seq analysis was performed using a SOLiD 3 Plus platform and an RNA-Seq kit (Life Sciences) according to the manufacturer's recommendations.

### *In silico* data analysis.

The sequencing data were analyzed using version 10.1 of CLC Genomics Workbench. The reads were trimmed on the basis of quality, with a threshold Phred score of 25. The reference genomes used for mapping were obtained from the NCBI database (H. capsulatum G186AR strain ABBS02 and G217B strain ABBT01). The alignment was performed using the following parameters: additional number of bases of upstream and downstream sequences, 100; minimum number of reads, 10; maximum number of mismatches, 2; nonspecific match limit, −2, minimum fraction length, 0.7 for the genome mapping or 0.8 for the RNA mapping. The minimum proportion of read similarity mapped on the reference genome was 80%. Only uniquely mapped reads were considered in the analysis. The libraries were normalized per million, and the expression values for the transcripts were recorded in RPKM (reads per kilobase per million). We also analyzed other expression values, including TPM (transcripts per million) and CPM (counts per million). The statistical test applied was the DGE (differential gene expression) test. For the ncRNA analysis, the database used was the ncRNA database from Histoplasma capsulatum (EnsemblFungi G186AR GCA_000150115 assembly ASM15011v1). The secondary structure analysis was performed using the PPFold plugin in CLC Genomics Workbench v. 10.1 and the default parameters. The entire RNA-seq database was subjected to PPFold analysis, and the putative structures were determined. Analysis of the relationship between the profile of RNA sequences detected in this study and the protein composition of H. capsulatum EVs was based on results recently obtained with strain G217B using a proteomic approach (25). The cellular RNA used in this analysis was assessed using the Sequence Read Archive (SRA) database (accession numbers SRR2015219 and SRR2015223) (28).

### Data availability.

The data were deposited into the SRA database under study accession number PRJNA514312.
